# Preliminary experience with cardiovascular magnetic resonance in evaluation of fetal cardiovascular anomalies

**DOI:** 10.1186/1532-429X-15-40

**Published:** 2013-05-21

**Authors:** Su-Zhen Dong, Ming Zhu, Fen Li

**Affiliations:** 1 Department of Radiology, Shanghai Children’s Medical Center, Shanghai Jiaotong University School of Medicine, Shanghai 200127, China; 2 Department of Cardiology, Shanghai Children’s Medical Center, Shanghai Jiaotong University School of Medicine, Shanghai 200127, China

**Keywords:** Fetus, Cardiovascular magnetic resonance, Congenital heart disease, Cardiac malformations, Prenatal diagnosis

## Abstract

**Background:**

The cardiovascular system is the part of the fetal anatomy that most frequently suffers from congenital pathology. This study shows our preliminary experience with fetal cardiovascular magnetic resonance (CMR) to evaluate congenital cardiovascular abnormalities.

**Methods:**

Between January 2006 and June 2011, Prenatal routine obstetric ultrasound (US), echocardiography and CMR data from 68 pregnant women carrying fetuses with congenital cardiovascular anomalies were compared with postnatal diagnoses (postnatal imagings, surgery and autopsy). All prenatal CMR was performed at 1.5 T. Imaging sequences included steady-state free-precession (SSFP) sequences, real-time SSFP and single-shot turbo spin echo (SSTSE) sequences. The images were analyzed with an anatomic segmental approach by two radiologists.

**Results:**

Fetal CMR yielded the same diagnosis as postnatal findings in 79% (54/68) of patients. The diagnostic sensitivity of routine obstetric US for cardiac anomalies was 46% (31/68). The diagnostic sensitivity of fetal echocardiographic examination by a fetal cardiac specialist was 82% (56/68). In 2 (3%) of 68 cases, diagnoses with both echocardiography and CMR were incorrect when compared with postnatal diagnosis. In ten (15%) cases, diagnosis at echocardiography was incorrect and that at CMR was correct. In twelve (18%) cases, diagnosis at echocardiography was correct and that at CMR was incorrect. Ten cases missed or misdiagnosed by echocardiography but correctly diagnosed by fetal CMR included asplenia syndrome (n = 2), interrupted inferior vena cava of polysplenia syndrome (n = 1), tricuspid incompetence (n = 1), double outlet right ventricle (n = 2), double aortic arch (n = 1), right pulmonary artery hypoplasia (n = 1), right-sided aortic arch of tetralogy of Fallot (n = 1) and hypoplastic left heart syndrome of a twin fetus (n = 1).

**Conclusion:**

Fetal CMR is a promising diagnostic tool for assessment of congenital cardiovascular abnormalities, especially in situations that limit echocardiography.

## Background

The cardiovascular system is the part of the fetal anatomy that most frequently suffers from congenital pathology [1]. The fetal heart is routinely studied using first level obstetric ultrasound (US). If a fetus is considered to have a cardiovascular defect, a detailed echocardiographic examination is required. The role of fetal cardiovascular magnetic resonance (CMR) as a tool additional to ultrasound has grown exponentially. Unlike ultrasound imaging, however, CMR is relatively unaffected by maternal and fetal conditions such as obesity, oligohydramnios and fetal complex malformations of various organ systems which particularly impair sonographic visualization of the fetal heart and great vessels. CMR avoids exposure to ionizing radiation and to the best of our knowledge, no clinical or experimental evidence has indicated that CMR has any adverse effects on the human fetus [2]. Recently, the successful development of new sequences during free breathing without cardiac triggering has established the beginning of a potential role for CMR in the study of the fetal cardiovascular system [3-5]. The literatures regarding the CMR diagnosis of fetal cardiovascular anomalies is composed essentially of case reports [6-8]. To the best of our knowledge, however, a large group of patients with a fetal cardiovascular abnormality diagnosed by CMR has rarely been reported either from a single center or as pooled data from multiple centers [9]. Here, we review 68 cases and report our preliminary experience with fetal CMR in the diagnosis of the cardiac and vascular malformations.

## Methods

### Study subjects

The ethics committee of Shanghai Children’s Medical Center approved this study. Written informed consent and the permission for imaging were obtained from all mothers. Between January 2006 and June 2011, 68 pregnant women carrying fetuses with cardiovascular abnormalities were evaluated using fetal echocardiography (Echo) and fetal CMR. The cases included three twin pregnancies in which one fetus was suffered from cardiovascular abnormality and 65 singleton pregnancies. Among these 68 cases, fetal CMR was performed at 20 to 36 weeks’ gestation (mean 25.5 weeks). The age of the pregnant women ranged from 22 to 38 years (mean 29 years). A detailed fetal echocardiographic examination by a specialist was usually performed within average 1.5 (1 to 2) days before or after the fetal cardiac MR examination. As regards extracardiac abnormalities, sixty-eight cases included one case with omphalocele, one case with gastroschisis, one case with diaphragmatic hernia, one case with oligohydramnios, two cases with polyhydramnios, one case with scoliosis and sixty-one cases not with other abnormalities affecting the diagnosis of the cardiovascular malformations. In all cases, CMR findings were compared with prenatal routine obstetric US, echocardiographic and postnatal diagnoses considered as standard of reference. Postnatal evaluation included postnatal imagings (echocardiography, late gadolinium enhancement CMR, cardiac enhanced CT and angiography), surgery and autopsy (Table 1).

**Table 1:** Postnatal evaluation methods to determine the final diagnoses

**No. of neonates**	**Methods**
7	Only echocardiography
6	CMR and echocardiography
4	Cardiac CT and echocardiography
2	Cardiac angiography and echocardiography
34	Surgery and echocardiography and CMR or CT
15	Only autopsy

### Magnetic resonance imaging

Between January 2006 and December 2010, prenatal CMR was performed using a 1.5-T unit (Signa Echospeed; GE Medical Systems, Milwaukee, WI, with 33 mT/m gradients) and an eight-channel phased array cardiac coil. After December 2010, prenatal CMR was performed using a 1.5-T unit (Achieva Nova dual; Philips Medical Systems, Best, The Netherlands, with 60 mT/m gradients) and an sixteen-channel sense-xl-torso coil. In order to minimize claustrophobia, the patients were placed in the magnet in a supine, feet-first position. Imaging sequences of GE included a fast imaging employing steady-state acquisition (FIESTA) sequence (TR/TE, 3.6/1.8 msec; field of view, 260–340 mm^2^; section thickness, 4–5 mm; spacing, 0–0.5 mm; matrix, 224 × 224–256 × 256; flip angle, 45–60°), a “real-time” non-gated FIESTA cine CMR sequence and a single-shot fast spin-echo (SSFSE) sequence (TR/TE, 1150–1450/90–135 msec; field of view, 250–300 mm^2^; section thickness, 4–6 mm; spacing, 0–0.5 mm; matrix, 256 × 160–256 × 192; flip angle, 90°). Imaging sequences of Philips included a balanced turbo field echo (B-TFE) sequence (TR/TE, 3.6/1.8 msec; field of view, 260–325 mm^2^; section thickness, 4–6 mm; spacing, -2–4 mm; matrix, 172 × 173–216 × 218; flip angle, 80°), a “real-time” B-TFE (B-TFE-RLT) sequence and a single-shot turbo spin echo (SSTSE) sequence (TR/TE, 12000/80 msec; field of view, 260–355 mm^2^; section thickness, 4–6 mm; spacing, 0–0.5 mm; matrix, 172 × 173–236 × 220; flip angle, 90°).

SSFP and SSTSE sequences provide T2-weighted images [5,10]. SSFP images were acquired in the transverse, four-chamber, short-axis, coronal and oblique sagittal views. SSTSE images were acquired only in an oblique coronal view in order to show the bronchus and for evaluation of the visceroatrial situs. Non-gated SSFP cine CMR was acquired in the transverse and short axis views. The total acquisition time was approximately 10–20 seconds per SSFP or SSTSE sequence, and the examination duration averaged approximately 20 min. No sedatives, intravenous gadolinium-based contrast media, or fetal cardiac gating were used.

### Image interpretation

All fetal MR examinations were performed and the images interpreted in consensus by two authors (S.Z.Dong and M. Zhu). S.Z. Dong had 10 years of experience in fetal US imaging and 8 years of experience in fetal CMR, M. Zhu had 30 years of experience in pediatric cardiac imaging and 8 years of experience in fetal CMR. All examinations were performed with prior knowledge of the first level obstetric ultrasonographic findings. The echocardiography results were available and known in 41 (60%) of 68 cases and not known in other 27 (40%) cases to the CMR radiologist at the time of acquisition and interpretation of MR images. But all postnatal results were not known to the CMR radiologist when MR images were interpreted.

For evaluation of MR images, the whole fetus including the cardiovascular structures and all other organ systems was assessed for anomalies using a standard protocol. Fetal cardiac structures were analyzed using a modified anatomic segmental approach of congenital heart disease (CHD) [3,4,11]:

(a) Visceroatrial situs: The stomach normally lies to the left and the liver and inferior vena cava to the right. Assessment of the situs in relation to the bronchial tree was attempted in each case. The embryologically normal left main bronchus is long with no early division and runs under the left pulmonary artery, whereas the right one is short, closer to vertical, has an early division and runs behind the right pulmonary artery.

(b) Position of the heart, cardiac apex and cardiac axis: Most of the heart normally lies in the left thorax with the cardiac apex to the left, only a small portion of the left atrium, about half the right atrium and a tiny corner of right ventricle lie in the right thorax. The normal cardiac axis lies at a 45°angle (range 22–75°) between the true sagittal plane (between the spine and the anterior chest wall) and a plane along the interventricular septum.

(c) Ventricular looping: In a normal D-loop, the more anterior ventricle is the morphologic right one with a trabeculated septal wall and the most posterior is the morphologic left one.

(d) Atrioventricular junction and cardiac chambers: In concordant AV connection, the right atrium connects to the morphologically right ventricle and the left atrium to the morphologically left ventricle. Seen in a balanced four-chamber view, the number of cardiac chambers, relative size of both atria and both ventricles were evaluated.

(e) Ventriculoarterial connections: Normally the aorta is connected to the morphologically left ventricle. The pulmonary artery normally is connected to a morphologic right ventricle and bifurcates at its distal end. The aorta can be traced in a regular arch that gives rise to three neck vessels. The main pulmonary artery crosses above the aortic root in a sagittal plane. The relative size of the aorta and pulmonary artery was evaluated.

(f) The systemic and pulmonary venous connections: Normally, both superior and inferior vena cava join the right atrium, and two right and two left pulmonary veins connect to the left atrium. The number of superior and inferior vena cava was evaluated.

(g) The following cardiac and other organ structures also were analyzed: Number and side of the aortic arch, intactness of interventricular septum, atrioventricular and great arterial valves, appearance and location of the liver and presence, appearance, and number of spleens.

## Results

### Frequency of confirmed fetal cardiovascular anomalies

**Figure 1:**
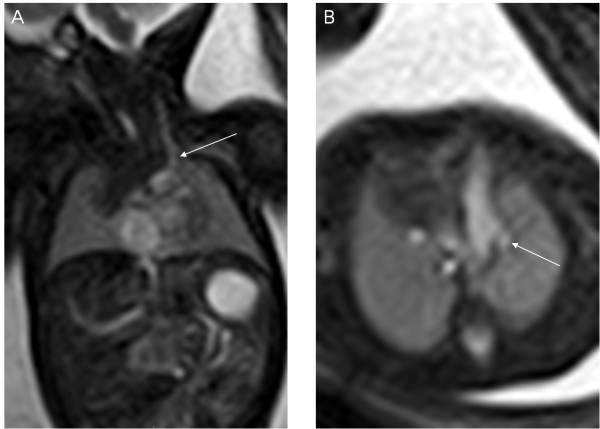
**A 33-week fetus with persistent left superior vena cava.** Fetal MR B-TFE coronal and transverse view images show persistent left superior vena cava (PLSVC) (arrows in **A** and **B**).

**Figure 2:**
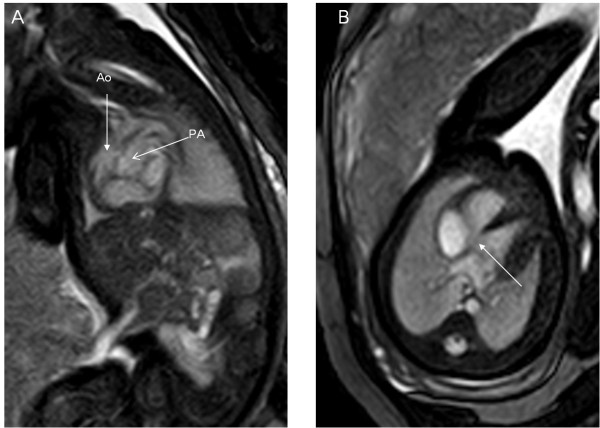
**A 34-week fetus with transposition of great arteries.** Fetal MR B-TFE oblique sagittal and transverse view images show parallel aorta (Ao) and pulmonary artery (PA) (arrows in **A**) and VSD (arrow in **B**).

**Figure 3:**
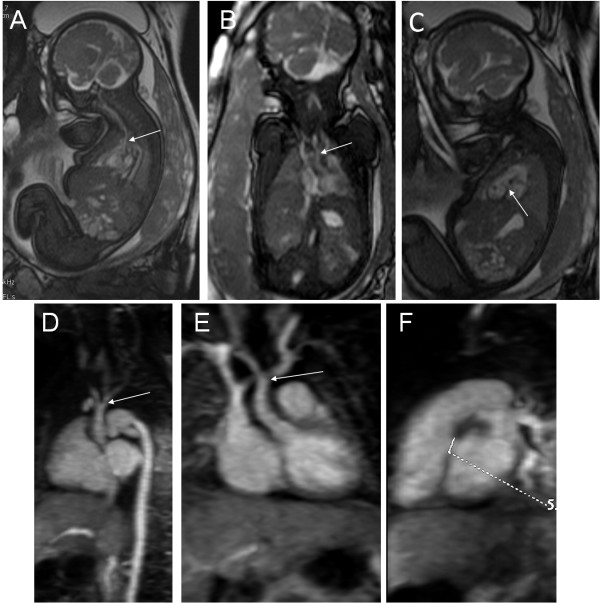
**A 34-week fetus, interruption of the aortic arch with VSD.** Fetal MR FIESTA oblique sagittal and coronal view images show the interruption of the aortic arch (arrows in **A** and **B**). MR FIESTA short-axis view image shows the VSD (arrow in **C**). Postnatal follow-up contrast-enhanced magnetic resonance angiography (CE-MRA) oblique sagittal and coronal view images show the interruption of the aortic arch (arrows in **D** and **E**). Postnatal CE-MRA short axis view image shows the VSD (measuring mark in **F**), compared to prenatal imaging.

**Figure 4:**
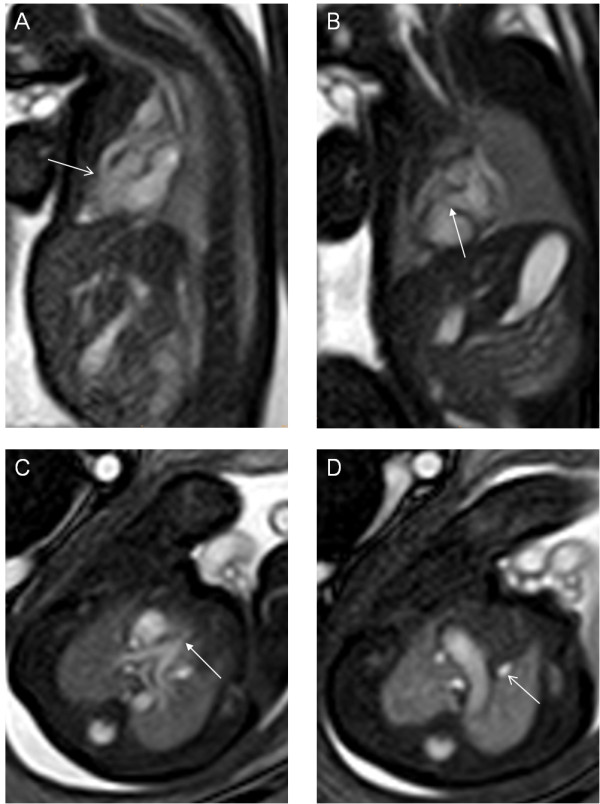
**A 28-week fetus, TOF with persistent left superior vena cava.** Fetal MR B-TFE oblique sagittal, short-axis, transverse view images show right ventricular outflow tract obstruction (arrow in **A**), small VSD (arrow in **B**), pulmonary stenosis (arrow in **C**) and persistent left superior vena cava (arrow in **D**).

The 68 cases of congenital cardiovascular anomalies included 23 categories of single or multiple cardiovascular defects. According to the anatomic segmental approach to congenital heart disease, fetal cardiac and vascular anomalies in this study involved atrial anomalies (n = 12) (Figure 1), atrioventricular and ventricular malformations (n = 18), ventriculo-arterial junction abnormalities (n = 14) (Figure 2), outflow vessels abnormalities (n = 15) (Figure 3) and other complex malformations (n = 9) (Figure 4) (Table 2).

**Table 2:** Frequency of confirmed congenital cardiovascular anomalies by means of postnatal final diagnosis (reference standard)

**Frequency of abnormalities of the cardiovascular system**	**No. of fetuses**
Situs inversus	2
Heterotaxy, cardiosplenic syndromes	8
Persistent left superior vena cava without other CHD	1
Atrial septal aneurysm	1
Atrioventricular canal defects	3
Tricuspid atresia	3
Tricuspid incompetence	4
Ebstein anomaly	1
Ventricular septal defect	5
Single ventricle	2
Double outlet right ventricle	9
Persistent truncus arteriosus	1
Transposition of great arteries	4
Coarctation of the aorta	2
Interruption of the aortic arch	3
Double aortic arch	1
Right-sided aortic arch without TOF	1
Pulmonary stenosis	1
Pulmonary atresia with VSD (PA/VSD)	1
Absent one pulmonary artery	1
Right pulmonary artery hypoplasia	5
Tetralogy of Fallot	6
Hypoplastic left heart syndrome	3

### Comparison between all diagnostic results

31 (46%) of 68 cases were correctly diagnosed with routine obstetric US when compared with postnatal diagnosis. In 44 (65%) of 68 cases, the diagnoses established by using echocardiography and CMR were correct when compared with postnatal diagnosis. In 54 (79%) of 68 cases, fetal MR examination yielded the same diagnosis as postnatal diagnosis. In 56 (82%) of 68 cases, fetal echocardiographic examination by a fetal cardiac specialist yielded the same diagnosis as postnatal diagnosis. In 2 (3%) of 68 cases, diagnoses with both echocardiography and CMR were incorrect when compared with postnatal diagnosis. The two cases included one case of tricuspid incompetence and one case of persistent truncus arteriosus (Table 3).

**Table 3:** Comparison between prenatal echocardiographic results, fetal CMR and postnatal findings

**Both echo and CMR incorrect**	**Echo correct, CMR incorrect**	**Echo incorrect, CMR correct**
TI (right cardiomegaly diagnosed at both)	Two atrioventricular canal defects (common atrioventricular valve missed at CMR)	Asplenia syndrome with omphalocele (cardiac structure not clearly showed at Echo)
Persistent truncus arteriosus (PA/VSD suspected at both)	*Two TA (absent TV not clearly showed at CMR)	Asplenia syndrome with CDH (RAoA, PLSVC, DAo and IVC on the same side missed at Echo)
	Ebstein anomaly (dilated right atrium diagnosed at CMR)	Polysplenia syndrome (interrupted IVC missed at Echo)
	Two small VSD (3 mm,missed at CMR)	*TI (thinned DA missed at Echo)
	CoA (mild, missed at CMR)	DORV with VSD, bilateral multicystic dysplastic kidney and oligohydramnios (DORV missed at Echo)
	PS (not clearly showed at CMR)	DORV with CAVC and polyhydramnios (DORV missed at Echo)
	*Two right pulmonary artery hypoplasia (hypoplastic pulmonary artery not clearly showed at CMR)	Double aortic arch (not clearly showed at Echo)
	TOF (overriding aorta not clearly showed at CMR)	Right pulmonary artery hypoplasia (only one scoliosis diagnosed at Echo)
		* TOF (right-sided aortic arch missed at Echo)
		HLHS of a twin fetus (cardiac structure not clearly showed at Echo)

* The main results of pretnatal diagnosis are almost consistent with postnatal findings. In 2 TA, The fetal MR images were analyzed to reach the diagnosis of TA by signs of reduced right ventricles. Two right pulmonary artery hypoplasia were detected essentially through the analysis of CMR signs of reduced right lungs; In 1 TI, the diagnosis of fetal echocardiography was correct, only thinned DA was missed. One case of TOF was correctly diagnosed by fetal echocardiography, only RAoA was missed by echocardiography.

TI: tricuspid incompetence; PA/VSD: pulmonary atresia with ventricular septal defect; TA: tricuspid atresia; TV: tricuspid valve; VSD: ventricular septal defect; CoA: coarctation of the aorta; PS: pulmonary stenosis; TOF: Tetralogy of Fallot; RAoA: right-sided aortic arch; PLSVC: persistent left superior vena cava; DAo: descending aorta; IVC: inferior vena cava; DA: ductus arteriosus; DORV: double outlet right ventricle; CAVC: complete atrioventricular canal; HLHS: hypoplastic left heart syndrome.

In twelve (18%) cases, diagnosis at echocardiography was correct and that at CMR was incorrect. In 2 (17%) of 12, tricuspid atresia (TA) was correctly suspected by CMR according to significantly reduced right ventricle, but absent tricuspid valve was not clearly showed at CMR. In 2 (17%) of 12, hypoplastic right pulmonary artery was not clearly showed at CMR, but right lung hypoplasia was correctly diagnosed by CMR. Other eight (67%, 8/12) cases included two cases of atrioventricular canal defects, one case of Ebstein anomaly, two cases of small ventricular septal defects (VSD, 3 mm), one case of coarctation of the aorta (CoA), one case of pulmonary stenosis (PS) and one case of the overriding aorta of tetralogy of Fallot (TOF) (Table 3). In twelve cases, fetal CMR was performed at gestational age from 22 to 26 weeks.

**Figure 5:**
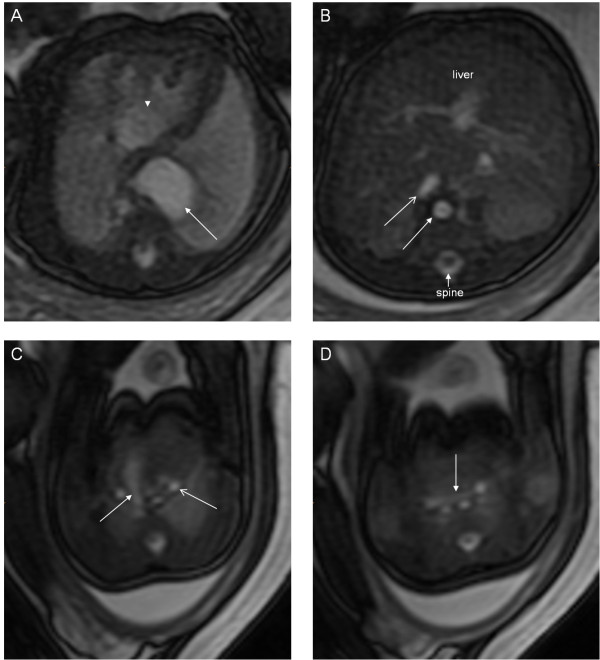
**A 35-week fetus, asplenia syndrome with diaphragmatic hernia.** Fetal MR FIESTA different level transverse view images show stomach in left side of chest (arrow in **A**) and heart deviated toward right (arrowhead in **A**), IVC (open arrow in **B**) anterior to descending aorta (DAo) (arrow in **B**) on same side of spine and midline liver (**B**), right-sided aortic arch (RAoA) (arrow in **C**), PLSVC ( open arrow in **C**) and bridging vein connecting the left and right superior vena cava (arrow in **D**).

**Figure 6:**
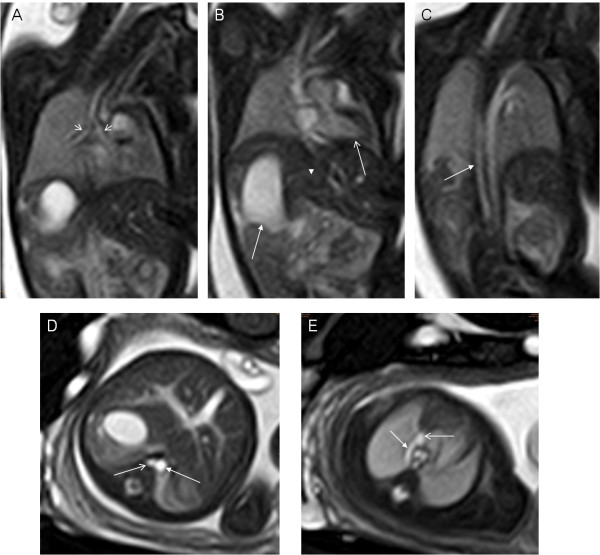
**A 28-week fetus with polysplenia syndrome.** Fetal MR B-TFE sequential coronal view images show bilateral main bronchial symmetry (open arrows in **A**), heart (open arrow in **B**) and stomach (right-sided, arrow in **B**) on opposite sides, midline liver (arrowhead in **B**), right azygos continuation (arrow in **C**). Fetal MR B-TFE transverse view images show azygos vein (open arrow in **D**) on right side of abdominal aorta (arrow in **D**), azygos vein arch (arrow in **E**) importing the superior vena cava (open arrow in **E**).

**Figure 7:**
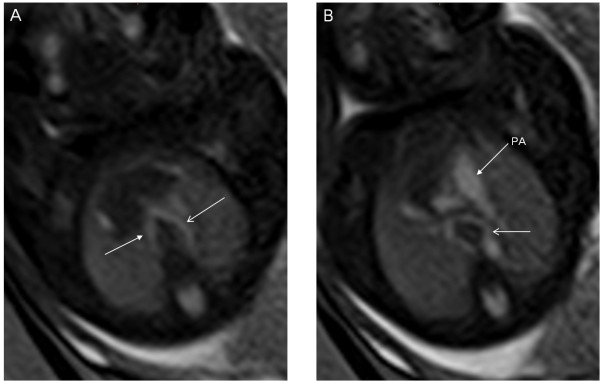
**A 26-week fetus with double aortic arch.** Fetal MR B-TFE sequential transverse view images show right-sided aortic arch (arrow in **A**), left-sided aortic arch (open arrows in **A** and **B**).

**Figure 8:**
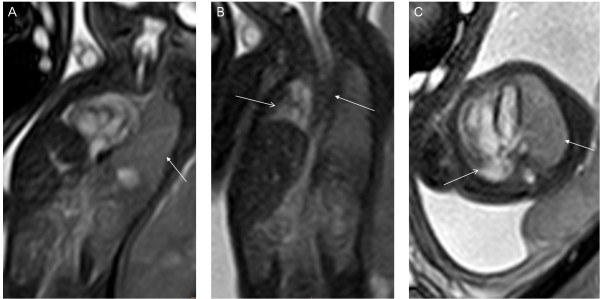
**A 27-week fetus with right pulmonary artery hypoplasia due to scoliosis.** Fetal MR B-TFE coronal and transverse view images show normal left lung (arrows in **A** and **C**), hypoplastic right lung (open arrows in **B** and **C**) and cervical scoliosis (arrow in **B**).

**Figure 9:**
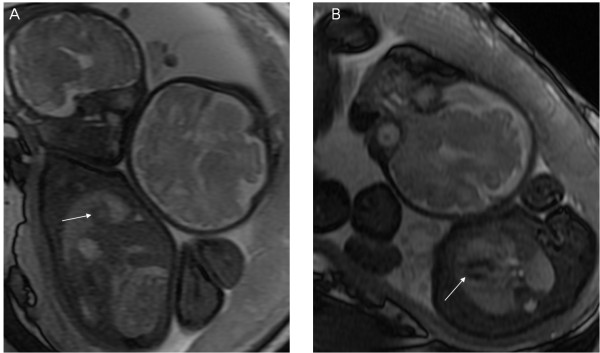
**A 32-week twin pregnancy, one with hypoplastic left ventricle syndrome.** Fetal MR FIESTA short-axis and transverse view images show the tiny left ventricle (LV) of the fetus far away from the uterine wall (arrows in **A** and **B**).

In ten (15%) cases, diagnosis at echocardiography was incorrect and that at CMR was correct. In 1 (10%) of 10, TOF was correctly diagnosed by echocardiography, only right aortic arch of TOF was missed. In 1 (10%) of 10, tricuspid incompetence (TI) was correctly diagnosed by echocardiography, only thinned ductus arteriosus was missed. Other eight (80%, 8/10) cases included two cases of asplenia syndrome (Figure 5), one case of interrupted inferior vena cava (IVC) of polysplenia syndrome (Figure 6), two cases of double outlet right ventricle (DORV), one case of double aortic arch (Figure 7), one case of right pulmonary artery hypoplasia (Figure 8) and one case of hypoplastic left heart syndrome (HLHS) of a twin fetus (Figure 9). Among ten cases incorrectly diagnosed by echocardiography, five cases were associated with extracardiac abnormalities that alter the anatomy of torso or abnormal amniotic fluid volume thus affecting the diagnosis of the cardiovascular malformations, one case was a twin fetus with 32-week gestational age. These extracardiac abnormalities or abnormal amniotic fluid volume included omphalocele (n = 1), congenital diaphragmatic hernia (CDH) (n = 1), bilateral multicystic dysplastic kidney and oligohydramnios (n = 1), polyhydramnios (n = 1) and scoliosis (n = 1) (Table 3). In the majority of ten cases, fetal echocardiography was performed at gestational age less than 22 weeks (n = 3) or greater than 28 weeks (n = 5).

## Discussion

Prenatal cardiac imaging is critical for the early diagnosis of congenital cardiovascular anomalies as well as for the management of the pregnancy in terms of counseling and delivery planning. CMR has now been confirmed as an important complementary tool to add to obstetric ultrasound for examining the fetal central nervous system, fetal non-central nervous system disorders, and placental and uterine diseases [12-15]. CMR has also emerged as a powerful approach to imaging congenital heart disease in neonates and infants. CMR avoids exposure to ionizing radiation, and at present, there is no evidence that short-term exposure to electromagnetic fields of 1.5 T or less harms the fetus [2,16,17]. CMR has the potential to expand its ability to image the fetal congenital cardiovascular abnormalities [9,18]. The very preliminary work presented here suggests that diagnoses of fetal congenital cardiovascular anomalies can be obtained in most patients using fetal CMR.

Although fetal echocardiography has been used successfully for many years in the diagnosis of fetal congenital cardiovascular abnormalities, there is a need at times for additional information without limitation to the acoustic windows [19]. The usual timing of fetal echocardiography for the premature diagnosis of congenital heart disease is at approximately 20–26 weeks of gestation. During the later phases of gestation, the development of certain conditions (e.g., relative reduction of the amniotic fluid volume and intensification of calcification in the ribs) may impair the quality of the fetal echocardiography examination [20]. In this study, in the majority of ten cases missed by echocardiography, fetal echocardiography was performed at gestational age less than 22 weeks or greater than 28 weeks.

CMR has no such limitations. CMR is also relatively unaffected by maternal and fetal conditions such as obesity, twins, oligohydramnios, polyhydramnios and fetal complex malformations of various organ systems [12,21], which particularly impair echocardiographic visualization of the fetal heart and vessels. Therefore, if structures cannot be visualized by fetal echocardiography, CMR can be considered as an alternative imaging modality. In this study, after suspected cardiac abnormalities or unclear cardiac structure with routine obstetric US or echocardiography, 68 fetuses have been referred for CMR to corroborate the diagnosis and search for associated extracardiac anomalies. The results showed that 50% (5/10) cases missed by echocardiography were associated with extracardiac abnormalities that alter the anatomy of torso or abnormal amniotic fluid volume thus affecting the diagnosis of the cardiovascular malformations, but the cases with extracardiac malformations were all correctly diagnosed by fetal CMR. In addition, a twin fetus with hypoplastic left ventricle syndrome, owing to large gestational age (32w) and twin pregnancy, was not clearly showed at echocardiography, but successfully diagnosed using fetal CMR. It is because this group of patients including many cases of complex heart defects and complex extracardiac malformations of various organ systems, the diagnostic accuracy of fetal echocardiography was slightly lower (82%).

As regards to technical matters, SSFP sequences produce good quality images of the fetal heart and vessels. On SSFP images, blood is characterized by a high signal intensity, thereby improving the blood-to-tissue contrast at the endocardial surface, and thus permitting the cardiac chambers and vessels to be imaged [3,18]. On non-gated cine MR images, the movements of the atria and the ventricles are reproduced as a video, thus allowing the cardiac kinetics to be evaluated, especially cardiac valve regurgitation and shunt which are not seen on the static SSFP images. However, the temporal and spatial resolution of the images on the non-gated cine SSFP sequence are usually not very good. If one was only to image with static SSFP, that is generally adequate for anatomic imaging. The most efficient technique for characterizing the fetal cardiac anatomy appears to be SSFP sequences used in conjunction with non-gated SSFP cine sequences. Radiofrequencies pose a potential safety risk for the fetus. In our examination, the ceiling of the specific absorption rate was 3.0 W/kg. We found that the flip angle could be changed in order to the control of specific absorption rate and that the image remained almost unchanged. With advances in CMR technology such as self-gating, real cardiac gating and metric optimized gating [22-24], both temporal and spatial resolution will improve. Accordingly, images will be considerably clearer, and take less time to acquire. In the future, CMR will become a more important diagnostic tool for fetal congenital cardiac and vascular abnormalities.

**Figure 10:**
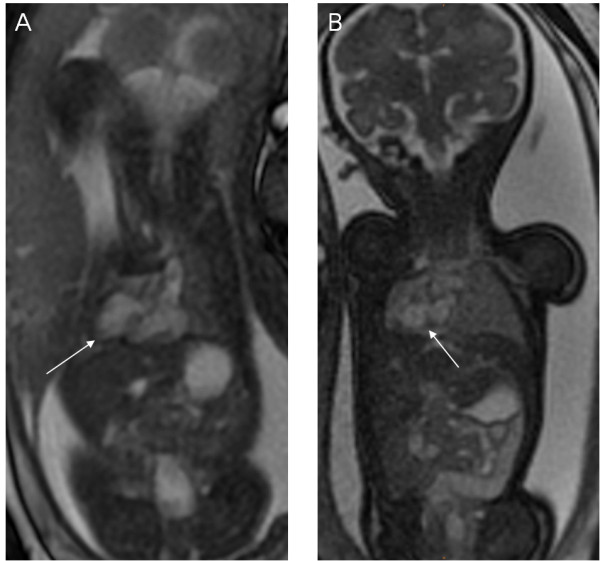
**The difference between dextrocardia and cardiac deviation, a 24-week fetus with dextrocardia (A) and A 33-week fetus with right lung hypoplasia (B).** Fetal MR FIESTA coronal view image shows heart (right-sided, with cardiac apex to the right) (arrow in **A**) and stomach on opposite sides, and heart (with cardiac apex to the left) deviated toward right owing to right lung aplasia (arrow in **B**).

Regarding diagnosis, the four-chamber view is very important. There are a number of congenital heart diseases (e.g., hypoplastic left heart syndrome, tricuspid atresia, single ventricle) that have a change of ventricular size. Prenatal CMR can diagnose these congenital heart diseases by the change in ventricular size in the four-chamber view. Prenatal CMR with its high tissue contrast is effective in demonstrating the fetal great vessels and associated anomalies (such as transposition of great arteries, interruption of the aortic arch, double aortic arch, right-sided aortic arch, persistent left superior vena cava). CMR provided additional important information to fetal echocardiography which confirmed and specified heterotaxy, cardiosplenic syndromes and complex malformation, in particular, CMR was valuable in the identification of unusual fetal extracardiac vascular anomalies such as persistent left superior vena cava and inferior vena cava interruption with azygous continuation, the latter a typical finding in polysplenia. Fetal CMR with its multiplanar capabilities is useful in the visualization of multiple spleens and confirmation of splenic absence, which might be related to poor survival outcomes. Sometimes fetal echocardiography is difficult to differentiate between true dextrocardia and extracardiac compression of a normal heart. Fetal CMR can show diaphragmatic hernia and one lung hypoplasia very clearly and can easily differentiate between dextrocardia and cardiac deviation (Figure 10). As in ultrasound examinations, it is important to have a radiologist who is familiar with congenital cardiovascular anomalies for the diagnosis of fetal cardiac and vascular malformations. Fetal CMR diagnostic accuracy rate (79%) is significantly higher than the routine obstetric US (46%) in this study.

When compared to echocardiography, fetal CMR does have some limitations. Fetal movements can affect imaging. Cardiac contractility and valvular functionality (atrioventricular and vascular) cannot be currently evaluated reliably with fetal CMR. CMR sometimes missed small ventricular septal defects and hypoplastic great vessels.

Our study has limitations. The number of cases of each pathology is small. Types of cases does not include pulmonary vein anomalies.

## Conclusions

In conclusion, CMR avoids exposure to ionizing radiation and is relatively unaffected by the conditions that impair echocardiographic visualization. Fetal CMR can be a promising diagnostic tool for assessment of congenital cardiovascular abnormalities, especially in situations that limit echocardiography. Our initial CMR results demonstrate that CMR is a useful adjunct in the assessment of fetal complex malformations of various organ systems (cardiac and extracardiac) and cardiosplenic syndromes. Further advances in technology will expand the role of fetal CMR in the evaluation of the fetal heart and vessels in the future.

## Competing interests

The authors declare that they have no competing interests.

## Authors’ contributions

SZD and MZ acquired the fetal MR data and optimised the MR sequences. All authors participated in the design and coordination of the study. SZD and MZ drafted the manuscript. All authors read and approved the final manuscript.
